# High serum concentrations of autoantibodies to HSP47 in nonspecific interstitial pneumonia compared with idiopathic pulmonary fibrosis

**DOI:** 10.1186/1471-2466-8-23

**Published:** 2008-11-04

**Authors:** Tomoyuki Kakugawa, Shin-ichi Yokota, Hiroshi Mukae, Hiroshi Kubota, Noriho Sakamoto, Syunji Mizunoe, Yasuhiro Matsuoka, Jun-ichi Kadota, Nobuhiro Fujii, Kazuhiro Nagata, Shigeru Kohno

**Affiliations:** 1 Second Department of Internal Medicine, Nagasaki University School of Medicine, Nagasaki, Japan; 2 Department of Microbiology, Sapporo Medical University School of Medicine, Sapporo, Japan; 3 Division of Pathogenesis and Disease Control, Department of Infectious Diseases, Oita University Faculty of Medicine, Oita, Japan; 4 Department of Molecular and Cellular Biology and CREST/JST, Institute for Frontier Medical Sciences, Kyoto University, Japan

## Abstract

**Background:**

The pathological diagnosis of idiopathic interstitial pneumonias (IIP) by surgical lung biopsy is important for clinical decision-making. However, there is a need to use less invasive biomarkers to differentiate nonspecific interstitial pneumonia (NSIP) from other IIP such as usual interstitial pneumonia (UIP). Heat shock protein (HSP) 47, a collagen-specific molecular chaperone, is involved in the processing and/or secretion of procollagen. HSP47 is increased in various fibrotic diseases. We investigated the autoantibodies to HSP47 in IIPs.

**Methods:**

We measured the serum levels of the autoantibodies to HSP47 in 38 patients with various forms of IIP [16 with idiopathic pulmonary fibrosis (IPF), 15 with idiopathic NSIP, 7 with cryptogenic organizing pneumonia (COP)] and 18 healthy volunteers.

**Results:**

The serum levels of autoantibodies to HSP47 in patients with idiopathic NSIP were significantly higher than in patients with IPF (P < 0.01), COP (P < 0.05), and healthy volunteers (P < 0.05). In addition, those in fibrosing NSIP were significantly higher than those of cellular and fibrosing NSIP (p < 0.05).

**Conclusion:**

We found high levels of anti-HSP47 autoantibody titers in sera of patients with idiopathic fibrosing NSIP compared with other IIPs and healthy volunteers.

## Background

The classification of idiopathic interstitial pneumonias (IIP) includes seven clinico-radiologic-pathological entities. Usual interstitial pneumonia (UIP) and nonspecific interstitial pneumonia (NSIP) are the two largest subsets of IIP [1,2]. The distinction between NSIP and UIP is important for clinical decision-making because the prognosis is generally better and the response to corticosteroids and immunosuppressants is also better in patients with NSIP compared with UIP [3-7]. In addition, patients with cellular NSIP are reported to have excellent long-term prognosis, while the majority of patients with fibrotic NSIP die mostly within 5 to 10 years of diagnosis [6]. Because of these reasons, the distinction between cellular NSIP and fibrotic NSIP is also important.

Clinicians often speculate on the presence of such pathological changes based on noninvasive imaging studies such as high-resolution computed tomography (HRCT) scans. However, the discrimination between NSIP and UIP cannot always be predicted accurately by HRCT. Although surgical (open or thoracoscopic) lung biopsy has been traditionally the ''gold standard'' for the diagnosis of interstitial lung diseases (ILD) and is clinically relevant for selection of appropriate therapy [8], it seems to be relatively invasive examination especially for patients with advanced ILD. Accordingly, less invasive biomarkers that distinguish NSIP from other types of IIP should be developed.

Heat shock protein (HSP) 47 is a collagen-binding, stress-inducible protein localized in the endoplasmic reticulum and is never released into the extracellular matrix. HSP47 has a specific role only in the intracellular processing of procollagen production as a collagen-specific molecular chaperone [9-12]. HSP47 expression is upregulated in animals with experimentally-induced fibrosis, including murine bleomycin-induced pulmonary fibrosis [13,14], rat peritoneal screlosis [15] and carbon tetrachloride-induced rat liver cirrhosis [16]. In addition, we reported previously that there was also increased expression of human HSP47 in the fibrotic lesions of idiopathic pulmonary fibrosis (IPF) [17,18], fibrotic transplanted kidney [19], and peritoneal sclerosis [20]. Recent reports have demonstrated that HSP47 expression is highly tissue- and cell-specific, restricted to mostly phenotypically altered collagen-producing cells, and correlates well with that of collagen [13,17-20]. These findings suggest the important role of HSP47 in collagen synthesis in various fibrotic disorders.

HSP47 is also identified as an autoantigen in the sera of several rheumatoid arthritis (RA) patients [21,22]. Higher levels of HSP47 protein and autoantibodies to HSP47 in sera were also found in patients with the rheumatic autoimmune diseases, especially mixed connective tissue disease (MCTD) [23]. Despite these observations, little is known about the relationship between fibrotic interstitial lung diseases and autoantibodies to HSP47.

We hypothesized that autoantibody titers to HSP47 in sera are different in idiopathic UIP, idiopathic NSIP, COP and healthy subjects.

## Methods

### Study populations

The subjects of this study were all the patients admitted to the hospitals of Nagasaki University School of Medicine and Oita University Faculty of Medicine from April 1997 to March 2004 in whom the diagnosis of interstitial pneumonia was confirmed pathologically, and 18 healthy adult volunteers. This is a retrospective study. The patients included 16 patients with IPF (UIP), 15 with idiopathic NSIP, 7 with cryptogenic organizing pneumonia (COP). The diagnosis of UIP and NSIP was confirmed pathologically by open lung biopsy or video-assisted thoracoscopic surgery (VATS) and classified according to the American Thoracic Society/European Respiratory Society consensus criteria [1]. Idiopathic NSIP patients included 7 with cellular and fibrosing pattern and 8 with fibrosing pattern [1]. The diagnosis of COP was established histopathologically by VATS in one patient and by transbronchial lung biopsy in 6 patients. Sera were obtained from these patients within one month before lung biopsy. Patients with fibrotic disease in any organ other than pulmonary fibrosis were excluded from the study, and no fibrotic disease other than pulmonary fibrosis was detected in any patient during the study. These patients had neither signs nor positive serological and other markers of collagen vascular diseases. Patients with cancer in any organ and those suspected to have malignancy were excluded from the study. None of these patients had received steroids or other immunosuppressants at the time of clinical sample collection. Patients characteristics before lung biopsy including age, smoking history, results of pulmonary function tests and arterial blood gas analysis were collected from either the clinical notes or the records of their general practitioners. Sera were also obtained from 8 healthy male and 10 healthy female volunteers (median age 31, range 26–60). All healthy volunteers had normal chest radiographs, were free of symptoms and not taking any medications. The study protocol was approved by the institutional review board, and informed consent was obtained from the patients and healthy volunteers.

### Determination of autoantibody titers by ELISA

Enzyme-linked immunosorbent assay (ELISA) for determination of autoantibodies to HSP47 was carried out essentially as described previously [23,24]. Briefly, recombinant HSP47 protein diluted at 1 μg/ml in 50 mM sodium carbonate buffer (pH 9.6) was immobilized on a 96-well microplate. The wells were blocked with 2% bovine serum albumin (BSA) in phosphate-buffered saline (PBS) and then incubated with human sera diluted 100-fold. Specific binding of serum IgG to HSP47 was detected by subsequent incubation of horseradish peroxidase-conjugated goat antibodies specific for the γ-chain of human IgG (BioSource, Camarillo, CA) and 3,3',5,5'-tetramethylbenzidine solution. After terminating the reaction with equal volume of 1 M phosphoric acid, absorbance at 450 nm was measured and used as an antibody titer.

### Statistical analysis

All values were expressed as median (range). Differences among groups were examined using Kruskal-Wallis test. The post-hoc test used was Scheffe test. Statistical analysis was performed using StatView-J 5.0 software (Abacus Concepts; Berkeley, CA). A *p *value < 0.05 denoted the presence of a statistically significant difference.

## Results

### Patient characteristics

Table 1 shows the characteristics of patients enrolled in this study. The baseline demographic and physiologic characteristics were similar among the groups.

**Table 1:** Patient characteristics

	COP	Idiopathic UIP	Idiopathic cellular and fibrosing NSIP	Idiopathic fibrosing NSIP	p value
Age (years)	68 (43–79)	64 (34–75)	71 (56–75)	50 (28–67)	ns
Sex (male/female)	5/2	11/5	2/5	4/4	ns
Smoking (none/ex/smoker)	3/3/1	6/4/6	5/1/1	4/2/2	ns
Spirometry:					
VC (L)	2.79 (1.86–4.21)	2.73 (1.14–3.60)	2.44 (1.28–3.32)	2.72 (1.73–3.39)	ns
predicted VC (%)	101.6 (53.0–111.2)	82.9 (43.3–112.5)	76.6 (56.8–108.1)	94.7 (66.9–101.5)	ns
FEV1 (L)	2.17 (1.39–2.81)	2.18 (1.04–2.67)	1.73 (0.99–2.53)	2.2 (1.26–2.98)	ns
predicted FEV (%)	77.6 (69.8–80.0)	83.3 (69.4–98.2)	85.0 (60.1–96.2)	80.9 (66.5–89.5)	ns
Gas exchange:					
DLco (ml/min/mmHg)	10.28 (6.39–17.81)	8.59 (2.32–14.65)	9.94 (7.24–14.60)	12.18 (7.77–19.85)	ns
predicted DLco (%)	58.6 (49.3–105.1)	46.3 (14.2–97.3)	67.5 (44.7–109.3)	53.1 (46.8–71.9)	ns
Lung volume:					
predicted TLC (%)	88.3 (49.0–98.4)	69.3 (32.6–78.7)	68.3 (66.3–104.2)	74.8 (62.9–96.6)	ns
TLC (L)	3.41 (2.32–5.81)	3.86 (1.27–4.78)	4.24 (2.27–8.80)	3.15 (2.44–4.46)	ns
Arterial blood gases:					
PaO_2 _(mmHg)	76.2 (61.3–83.1)	80.7 (47.2–103.3)	74.2 (70.0–86.4)	85.4 (77.5–92.9)	ns

Data are median (range). ns = not significant

COP = cryptogenic organizing pneumonia; DLco = diffusing capacity for carbon monoxide; FEV = forced expiratory volume; NSIP = nonspecific interstitial pneumonia; TLC = total lung capacity; UIP = usual interstitial pneumonia; VC = vital capacity.

### Anti-HSP47 autoantibody titers in human sera

**Figure 1:**
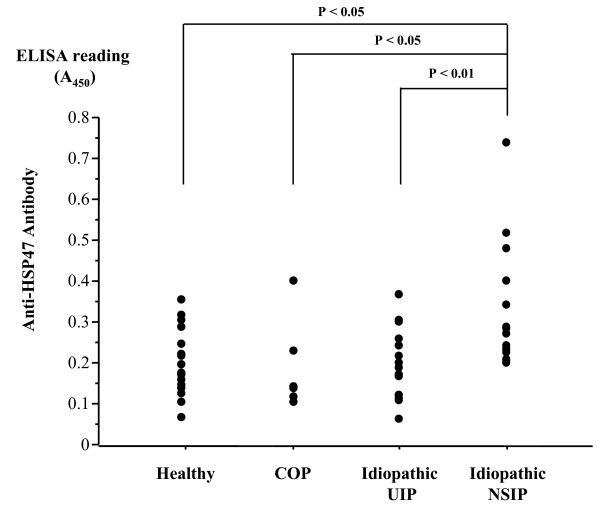
**Scattergram of IgG titers to HSP47 in patients with cryptogenic organizing pneumonia (COP), idiopathic usual interstitial pneumonia (UIP), idiopathic nonspecific interstitial pneumonia (NSIP) and healthy volunteer. **Antibody titers are expressed as absorbance at 450 nm.

**Figure 2:**
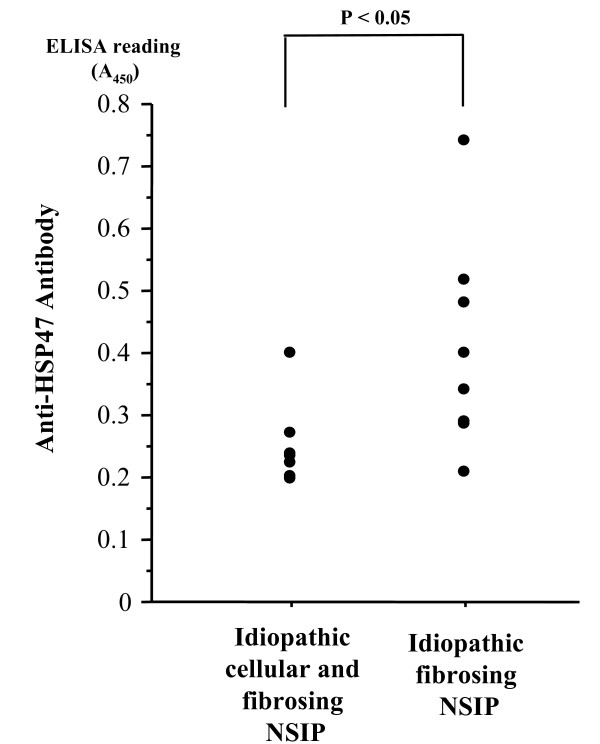
**Scattergram of IgG titers to HSP47 in patients with idiopathic cellular and fibrosing nonspecific interstitial pneumonia (NSIP) and idiopathic fibrosing NSIP.** Antibody titers are expressed as absorbance at 450 nm.

HSP47-reactive IgG titers of patients with idiopathic NSIP (median, 0.281 [range, 0.194–0.734]) were significantly higher than those of idiopathic UIP (0.165 [0.059–0.361]) (P < 0.01), COP (0.137 [0.101–0.394]) (P < 0.05), and healthy volunteers (0.181 [0.062–0.349]) (P < 0.05) (Fig. 1). HSP47-reactive IgG titers of patients with fibrosing NSIP (0.367 [0.206–0.734]) were significantly higher than those of the cellular and fibrosing NSIP (0.231 [0.194–0.395]) (P < 0.05) (Fig. 2). HSP47-reactive IgG titers of patients with cellular and fibrosing NSIP, UIP, COP and healthy controls were not significantly different.

### Correlation between anti-HSP47 autoantibody titers in sera and clinical course and other markers

Anti-HSP47 autoantibody titers did not correlate with survival or clinical data such as results of pulmonary function tests and arterial blood gas analysis (data not shown). Antibody titers to HSP47 also did not correlate with the serum levels of KL-6, surfactant protein (SP)-D and SP-A (data not shown).

## Discussion

The present study of biopsy-proven cases clearly demonstrated that the anti-HSP47 titers of patients with idiopathic fibrosing NSIP were higher than those of patients with idiopathic UIP, idiopathic cellular and fibrosing NSIP, COP and healthy subjects. In contrast, the anti-HSP47 autoantibody titers in sera of patients with idiopathic UIP and healthy controls were not different despite the fact that overexpression of HSP47 has been reported in fibrotic lesions of IPF patients [17,18].

Our results suggest that anti-HSP47 autoantibody titers in sera might be useful to discriminate between idiopathic fibrosing NSIP and other types of IIP such as IPF. However, we do not recommend the use of this serum marker alone to differentiate idiopathic fibrosing NSIP and other types of IIP because (1) our study only included a relatively small number of patients, (2) there was some overlap in the serum levels of anti-HSP47 autoantibodies between UIP and NSIP, (3) this is a retrospective study. Moreover, the patients groups might not represent general patient population with IIP because the diagnosis of UIP and NSIP was confirmed pathologically by open lung biopsy or video-assisted thoracoscopic surgery and patients diagnosed only clinically were excluded. Although surgical lung biopsy has been traditionally the "gold standard" for the diagnosis of ILD, it seems to be relatively invasive examination especially for patients with advanced ILD. Accordingly, the study population may represent only "early and moderate" stage of ILD. Well-planned prospective study using a large number of patients are required to determine the cutoff levels of anti-HSP47 autoantibody titers necessary for the diagnosis, together with analysis of the sensitivity and specificity of such levels.

In the present study, we also investigated whether serum titers of anti-HSP47 autoantibody correlated with clinical course and other serum markers. However, anti-HSP47 autoantibody titers did not correlate with survival or clinical data such as results of pulmonary function tests and arterial blood gas analysis (data not shown). This is probably because of the small sample size. In addition, the titer of HSP47 antibody did not correlate significantly with the serum levels of KL-6, SP-D and SP-A, which were previously reported to be correlated with clinical activity of interstitial pneumonia [25-27], probably because of differences in the origins of these markers.

Why were the serum levels of autoantibody to HSP47 elevated in idiopathic NSIP patients, but not in idiopathic UIP patients, while fibrotic changes and expression of HSP47 in the lungs of patients with idiopathic UIP are much more severe than those with NSIP [18]? HSP47 is usually never released into the extracellular matrix, so it appears that intracellular HSP47 protein leaks into the peripheral blood when there is inflammation leading to tissue destruction. Elevation of anti-HSP47 autoantibody titers in idiopathic fibrosing NSIP patients might be due to the distinctive characteristics of idiopathic fibrosing NSIP including a variable degree of inflammation and fibrosis within the alveolar walls, which may induce leakage of HSP47 protein into the peripheral blood and subsequently induce the production of anti-HSP47 autoantibody. In contrast, severe fibrosis is seen but inflammation is mild in idiopathic UIP. A previous report reported that MCTD patients had markedly high levels of both HSP47 protein and autoantibodies to HSP47 in the sera compared with other rheumatic autoimmune diseases, including rheumatoid arthritis, systemic lupus erythematosus, and Sjögren syndrome [23]. This also suggests that the combination of inflammation and fibrosis is necessary for HSP47 protein to leak into the peripheral blood. However, this theory does not account for the reason why HSP47-reactive IgG titers of patients with fibrosing NSIP were significantly higher than those of idiopathic cellular and fibrosing NSIP. Many of the patients with idiopathic NSIP develop collagen vascular disease later on in the disease course. The fact that the serum levels of autoantibodies to HSP47 in patients with idiopathic NSIP were significantly higher than in patients with other IIPs suggests that the patients with so called "idiopathic" NSIP might display signs of auto-immunity in contrast to patients with idiopathic UIP.

As we previously reported, expression of HSP47 was noted in fibroblasts, myofibroblasts and type II pneumocytes in idiopathic interstitial pneumonia [17,18]. The expression level of HSP47 in type II pneumocytes of idiopathic UIP was significantly higher than that in idiopathic NSIP [18]. In contrast to that, in this study, we demonstrated that the anti-HSP47 titers of patients with idiopathic NSIP were significantly higher than those of patients with idiopathic UIP. We speculate that HSP47 autoantibody might neutralize the HSP47 antigen and suppress the fibrosis in idiopathic NSIP. However, there is no direct evidence. Further studies are warranted in order to elucidate the precise mechanisms.

Nevertheless, our findings support the concept that these diseases are different pathophysiological entities with different fibrotic pathways. We speculate that evaluation of anti-HSP47 autoantibody titers might be a useful method to understand the differences in the underlying pathogenic mechanisms of these diseases.

## Conclusion

In conclusion, we found high levels of anti-HSP47 autoantibody titers in sera of patients with idiopathic fibrosing NSIP compared with idiopathic UIP, idiopathic cellular and fibrosing NSIP, COP and healthy volunteers. However, further studies of a large number of patients are required to determine the prognostic and therapeutic values of anti-HSP47 autoantibody titers.

## Competing interests

The authors declare that they have no competing interests.

## Authors' contributions

All authors read and approved the final manuscript. TK, SY and HM have made substantial contributions to conception and design. TK and SY have made substantial contribution to acquisition and analysis of data. SY have made substantial contribution to determination of autoantibody titers by ELISA. HK and YM have made substantial contribution to preparation of recombinant HSP47 protein. TK, SY, HM, NS, SM and JK have been involved in collecting clinical samples. TK, SY and HM have been involved in drafting the article. NS, NF, KN and SK have been involved in revising it critically for important intellectual content.

## Pre-publication history

The pre-publication history for this paper can be accessed here:


